# Effects of Food-Additive-Information on Consumers’ Willingness to Accept Food with Additives

**DOI:** 10.3390/ijerph15112394

**Published:** 2018-10-29

**Authors:** Yingqi Zhong, Linhai Wu, Xiujuan Chen, Zuhui Huang, Wuyang Hu

**Affiliations:** 1 School of Economics, Zhejiang Gongshang University, Hangzhou 310018, China; zhongyingqi@zjgsu.edu.cn; 2 Institute for Food Safety Risk Management & School of Business, Jiangnan University, Wuxi 214122, China; xjchen@jiangnan.edu.cn; 3 China Academy of Rural Development, Zhejiang University, Hangzhou 310058, China; zhhuang@zju.edu.cn; 4 Department of Agricultural, Environmental, and Development Economics, The Ohio State University, Columbus, OH 43210, USA; Hu.1851@osu.edu

**Keywords:** food safety, willingness to accept (WTA), food additives, random *n*th-price auction

## Abstract

This study tested whether information on positive food additives and negative food additives had an effect on consumers’ risk perception and their willingness to accept (WTA) food with additives. Consumers’ WTA was examined via a random *n*th-price auction of exchanging freshly squeezed orange juice without additives for orange juice with additives. Results show that consumers’ WTA differs with the order in which information was provided. Consumers are generally more sensitive to negative than positive information on additives. Female, middle-educated consumers are more susceptible to additive information and their WTA is more likely to change, while postgraduate-educated consumers are less sensitive to additive information. Consumers with higher food-safety satisfaction have lower WTA than those who are not satisfied with food safety. However, their satisfaction is easily affected by the negative-information intervention. Interestingly, consumers with relatively good knowledge of additives had higher WTA than those with no such knowledge. This study provides insight on how to establish effective food-safety-risk communication. Government and non-government agencies need to timely and accurately eliminate food-safety scares through the daily communication and disclosure of food-safety information, as well as prevent the misguidance of negative food safety-risk information.

## 1. Introduction

Food additives are the basis of the modern food industry, and play an important role in improving the color, smell, and taste of food, altering its nutritional structure, perfecting its processing conditions, and extending its shelf life [1,2]. Although the use of additives during food processing has become common practice on a global scale, consumers’ concerns about their potential risks have not abated [3,4]. In recent years, with continuous improvement in consumers’ quality of life, the demand for all-natural, no-additive food has kept increasing. As a result, a number of synthetic food additives have been included in the list of potential food-safety hazards, and many customers believe that the use of food additives is actually unnecessary or unwarranted [5,6].

In China, food-safety issues that are related to food additives, such as their misuse or overuse due to anthropogenic stressors, or the reckless use of nonedible chemical substances, have become increasingly prominent. Studies have shown that, between 2006 and 2015, a total of 253,617 food-safety incidents were reported in China, of which 75.5 percent were caused by anthropogenic factors. Moreover, the highest number of food-safety incidents was caused by the illegal use of food additives, which accounted for 34.36 percent of the total [7]. Indeed, the latest information that has been released by China’s State Food and Drug Administration revealed that, out of 257,000 batches of food samples that were collected nationwide, there were 8224 batches of substandard food, 33.6 percent of which was caused by the misuse or overuse of food additives by food production and processing organizations (http://www.sda.gov.cn/WS01/CL1199/168583.html). What is worse, some illegal enterprises used nonedible chemical substances in order to pursue their economic interests, which have caused real or potential damage to consumers’ health. A typical example is when an infant formula was contaminated with melamine, which occurred in October 2008 and resulted in 296,000 infants and young children having varying degrees of a urinary-system abnormality. The social impact of this incident was devastating in China, and, as a result, many customers have become increasingly worried about the safety of food additives [8]. Indeed, there is a great amount of evidence that has repeatedly shown that food-safety incidents occur frequently in China, which is mainly due to the abuse of food additives (The abuse of food additives refers to the practice of misuse or overuse of food additives, as well as the use of fake, shoddy, or expired food additives.) by food-production operators, whose goal is to pursue economic benefits regardless of their moral duty. It is therefore not surprising that such illegal practices have become one of the most worrying aspects of food-safety risks in the country [9].

According to the existing classification system in China, there are 23 categories and over 2000 varieties of food additives. Such complexity in the diversity and functionality of food additives makes it very difficult for consumers to comprehensively understand food additives. Indeed, even food scientists and engineers may not have a clear understanding of the subject. Due to the limitations of knowledge, false or negative information about food additives has been widely considered as correct ones. Additionally, some illegal additives or nonedible chemical substances are wrongly categorized as food additives, which can cause further harm. As a result, when information is dominated by the potential risks of food additives, authentic or scientific information is often in a disadvantageous position, and this can lead to customers worrying too much about the hazards of food additives [10], and even cause food scares. Against this background, this study tries to investigate the impact of orange-juice additive information on customers’ risk perception and their willingness to accept (WTA). In particular, the study uses a random *n*th-price auction to evaluate consumers’ willingness to accept some form of compensation when they exchange freshly squeezed orange juice without additives for orange juice containing additives. We used a Tobit model to examine whether the intervention of positive and negative information that is related to orange-juice additives had an effect on consumers’ WTA, as well as the main factors that influence their WTA.

## 2. Literature Review

Food properties in terms of its quality and safety are a special commodity and are therefore difficult to determine before consumption. Based on such properties, the concept of food-safety risk perception denotes people’s awareness of the potential risks or hazards of food quality and safety, and the judgment process regarding the serious consequences of the information and the likelihood and extent of its acceptance by the general public [11,12,13]. Although it is difficult to observe food-safety risk perception directly, consumers’ risk perception of a particular type of food would affect their food preference, and determine their willingness to pay (WTP) [14]. Thus, the perception of food-safety risk has great consequences on consumers’ WTP. Since consumers’ perception of food-safety risks could reduce the utility level of a typical food, a certain amount of compensation is needed to offset the corresponding value loss [15]. Consequently, WTA is better than WTP to quantify consumers’ preference of risky food, and infer their food-safety risk perception [16]. Furthermore, compared with WTP, WTA is less susceptible to the influence of subjective values and more sensitive; thus, it quickly corresponds to the market anchor price (The market price anchor is the reference point for consumers when they need to estimate the value of a commodity. As far as WTA is concerned, consumers are more likely to consider the market price as the reference point for the estimated value.) [17]. Some studies on consumers’ WTA have considered the influence of consumers’ risk perception. For example, Lusk et al. [18] applied the method of five-point auctions to study the preferences and WTA of consumers from the United States, the United Kingdom, and France in terms of genetically modified (GM) food. Ward, Bailey, and Jensen [19] quantified the magnitude of changes in consumers’ WTA regarding traceable beef following the release of information about mad-cow disease.

Food safety risk perception refers to people’s perception of the potential risks or hazards of food quality attributes. Consumers make judgements of purchasing based on such perceptions. Since consumers are not food experts and they do not have the expertise to accurately identify the health effects of purchased foods before or after consumption, it is not possible to determine the potential for health damage. Therefore, most consumers rely on intuition and experience to subjectively assess food safety risks. Consumers’ perception of the risk of food safety is different from the actual risk [20,21]. Consumers’ intuition and experience in assessing food safety risks stems primarily from their common sense in food safety, certain knowledge of the food and evaluation of current food safety governance environment [22]. For food additive in China, consumers lack knowledge about food additives, and the risk perception of food additives is easily interfered by external information, due to the complexity of it. Existing studies have confirmed that positive or negative information about food can affect customers’ risk perception, and further determine their attitude and buying behavior. Studies that are related to food-market consumption have found that negative information can lead directly to a reduction in food demand [23,24,25]. In recent years, the impact of positive and negative information concerning food-safety risks on consumers’ WTP has been investigated in many studies. For example, Fox, Hayes, and Shogren [26] examined the effects of favorable and unfavorable description of irradiation on consumers WTP for pork products under irradiation. They proved that favorable description increased WTP, while the unfavorable description decreased it; when consumers were given both descriptions, the negative description dominated. Tegene et al. [27] studied consumers’ WTP for genetically modified-labeled and standard-labeled foods under different information regimes. The result showed that consumers have lower WTP for the food labeled as genetically modified, and were more affected by negative information. Payne, Messer, and Kaiser [28] analyzed consumers’ WTP for the beef hamburger under the information of the BSE crisis, and found that consumers discounted beef products by an average of 59 percent under negative information. Lee et al. [29] compared the effect of positive, negative, and two-sided information of traceability on consumers’ WTP for imported beef. The result indicated that negative and two-sided information significantly reduced WTP, while the effect of positive information was insignificant. Such studies have suggested that consumers place a higher weight on negative information, and that WTP is significantly influenced by negative information, rather than positive information.

Studies have shown that negativity bias (Negativity bias, also known as negative effect, means that negative events (e.g., unpleasant thoughts, feelings, or social encounters, or harmful or traumatic events) have a greater impact on a person’s mental state and processes than neutral or positive events, even with the same intensity of stimulation.) exists in consumer food-safety risk perceptions [30,31]. Negativity bias leads to overestimating the certainty and probability of impossible events, as well as an increased vulnerability to negative information during information communication [32]. Therefore, distributing negative information and rumors related to food safety not only leads to wrong or misleading messages being sent to information seekers, but also aggravates the uncertainty of food-safety information. Indeed, the widespread dissemination of negative or false information related to food-safety risks could even interfere with consumers’ rational understanding of food-safety incidents [33], and aggravate their perception of food-safety risks.

Overall, the existing literature focuses on the risks of food produced with new technologies. For example, the majority of research focuses on risk perception and consumers’ preferences regarding genetically modified food, irradiated food, and other types of food produced via new technologies. With regard to research methodology, although some related studies use consumers’ WTA, most focus on consumers’ WTP. Studies that address consumers’ risk perception regarding food additives are rarely conducted, and only a handful of studies have been conducted that try to understand consumers’ perception of food safety risks through the estimation of their WTA.

## 3. Materials and Methods

### 3.1. Auction Design

#### 3.1.1. Choice of Auction Mechanism

The effectiveness of experimental auctions depends on the choice of auction mechanism. At present, the Vickrey [34], Becker-DeGroot-Marschak (BDM) [35], and random *n*th-price [36] auctions are the most widely used experimental mechanisms. The Vickrey auction is commonly used to measure consumer WTP for foods with different quality and safety information. In repeated Vickrey auctions, participants with a low valuation may lose interest in the auction, because they have no chance of winning, thus resulting in insincere bidding. As a result, a bidder’s valuation cannot be accurately revealed [37]. A BDM auction is a bid between a participant and randomizer. Each participant has a chance of winning, thus avoiding insincere bidding [35]. However, due to the lack of a competitive market environment, a BDM auction might not achieve incentive compatibility [38]. When compared with other auction mechanisms, the most basic feature of a random *n*th-price auction is incentive compatibility. It combines the advantages of the Vickrey and BDM auctions. Moreover, it produces an endogenous, clear market price during the auction, which ensures that the market price derived from the experimental auction is closely related to the individual valuation provided by the participant. Valuations obtained from the participants are unbiased and precise, thus overcoming the competitive bias of a Vickrey auction [36]. Because this study deals with human subjects, we have formal approval from the Ethical Committee of the Jiangnan University, China, before we actually conducted the experiments. Related documents have been examined by the journal in the review process.

#### 3.1.2. Auction Object and Experimental Location

The object of the experimental auction was orange juice containing additives. There were a number of reasons why orange juice was chosen. Firstly, orange juice that is sold on the market often contains several types of food additives, such as sweeteners, acidulants, colorants, thickeners, stabilizers, and preservatives, and the use of these food additives can help to maintain the sweetness, acidity, taste, and nutrients of orange juice, and extend its shelf life. Secondly, as one of China’s most popular soft drinks, fruit juice products have attained a large market share. Currently, China’s per capita consumption of orange juice is close to one kilogram. Thirdly, it is relatively easy to distinguish orange juice sold in the market from freshly squeezed orange juice by establishing whether food additives are added or not. Finally, since squeezing fresh orange juice is with high operability, which could help increase the credibility of experimental auctions and restore the real market environment, the use of orange juice as the object of experimental auctions has some unique advantages.

This experiment was performed in Suzhou City, Jiangsu Province. As Suzhou is a relatively developed city in China, in 2017, GDP in Suzhou was 16,555 billion yuan, ranking seventh in Chinese cities. Consumers in Suzhou are more aware of issues related to food safety, including the safety of food additives. Furthermore, orange juice is the most popular soft drink in Suzhou. To ensure representativeness of the samples, the participants were recruited from large supermarkets in four districts in the north, south, east, and west of Suzhou by posted advertisements and verbal notification. Participation was completely voluntary. Participants were then transported to a laboratory in Suzhou University for the experiment. At the time of recruiting, participants were informed that they would receive a reward of 50 CNY. No further information about food additives was provided in order to avoid systematic nonparticipation bias associated with food additives and food safety [39]. To facilitate understanding, PowerPoint presentations and posters were used to explain the experiment. Before the experiment, participants were asked several questions to ensure that they understood the experiment correctly.

#### 3.1.3. Steps of Random *n*th-Price Auction

The experiment was repeated eight times in total. Participants were divided into Groups A and B. Group A was first provided with positive information on orange juice additives and then negative information. Group B was provided with information in the opposite order. Nine bidding trials were conducted for Groups A and B, respectively, with reference to Hayes et al. [40], and Fox, Hayes, and Shogren [26]. In the first three bidding trials, no information was provided to either group. Group A participants were given positive information on orange-juice additives before the fourth trial, and negative information before the seventh trial, whereas Group B participants were provided with information in the opposite order. The positive information of the additive provided in this study is focused on its positive role in food technology objectively, and that food additives have good effects in facilitating people’s lives and enhancing people’s consumption experience. The negative information about the additive is also objective hazards that have been confirmed in extant studies. The bid could be less than or equal to zero, indicating that the participants do not think orange juice containing additives is any different than freshly squeezed orange juice in safety or taste. Table 1 shows the detailed recruitment plans of the experiment and Table 2 shows the positive and negative information provided during the auction.

The steps of the random *n*th-price auction were as follows:

Step 1: Each participant was given an identification number and asked to complete a short questionnaire dealing with their beliefs about food additives and some demographic information.

Step 2: In order to familiarize the participants with the random *n*th-price auction, a practice auction was conducted. We used a candy bar to train the participants on how the auction mechanism works. In this step, we made sure that all the participants absolutely understood the random *n*th-price auction, and their best strategy was to bid on their true valuation for the target product being auctioned.

Step 3: At the beginning of this step, two types of orange juice were shown to the participants. The freshly squeezed orange juice without additives was provided to the participants for free and the orange juice with additives was the target of this auction. The two types of orange juice were provided in identical clear plastic containers of the same volume. After introducing these two items, we let participants use the orange juice without additives to trade with the juice containing additives, and wrote down their WTA on the sealed biding sheet.

Step 4: The monitor collected the bids and reordered them from low to high. Then, the monitor randomly drew a number n from 2–*K* (*K* represents the number of participants). The *n*th participants’ bid $p_{n}$ would be the binding price. The winners were the subjects whose bids were under the *n*th bid. ID numbers of the winners and their corresponding bids were then announced to all participants.

Step 5: After conducting three auction trials, positive (negative) information about additives was provided. Then, before the last three trials, negative (positive) information was provided.

Step 6: After nine trials were concluded, a binding trial was randomly chosen. The winners in that trial had to exchange their orange juice with the alternative orange juice and accept the compensation determined in that trial (*n*th bid, $p_{n}$).

### 3.2. Model Building

#### 3.2.1. Tobit Model

To further analyze the factors involved in consumers’ WTA of the orange juice with additives, we defined the utility of a customer consuming one unit of freshly squeezed juice without additives and one unit of orange juice with additives as $U_{fki}$ and $U_{aki}$, respectively, with k=+,− representing positive and negative information. Thus:

(1) $$U_{fki}-U_{aki}=\beta_{k}^{T}x_{i}+\varepsilon_{ki}$$

where $\beta_{k}$ is the parameter vector; $x_{i}$ is the factor effect on consumer utility, e.g., demographic characteristics of consumers, understanding of additives, and risk perception of additives; and, $\varepsilon_{ki}$ is the random vector. Although $U_{fki}$ cannot be obtained directly, it can be derived from the exchange auction. According to the definition of WTA, we can suppose that the WTA of consumer i is $WTA_{ki}$, which represents the minimum compensation of consumer i to change from freshly squeezed orange juice without additives to orange juice containing additives. Thus:

(2) $${WTA}_{ki}=\beta_{k}^{T}x_{i}+\varepsilon_{ki}$$

According to Function (1), there are two possibilities of $WTA_{ki}$, that are $WTA_{ki}>0$ and $WTA_{ki}\leq 0$. When $WTA_{ki}\leq 0$, Function (2) suggests that consumer i preferred orange juice with additives rather than orange juice without additives, or consumers think they are identical. When consumer preferred orange juice with additives, they are unwilling to use orange juice containing additives in exchange for orange juice without additives. Thus, in order to consume orange juice with additives, they are willing to pay some money to retain the juice with additives. Under this circumstance, their WTA was below zero. Here, $WTA_{ki}$ is equal to WTP.

Assuming $\varepsilon_{k}|x_{k}\sim Normal(0,\sigma_{k}^{2})$, Function (2) is a multiple linear regression. When $WTA_{ki}\leq 0$, consumer i is unwilling to accept orange juice without additives. Therefore, the Function can be transformed into Tobit model. The Tobit model can be described, as follows:

(3) $$y_{ki}=\{\begin{array}{cc} WTA_{ki} & WTA_{ki}>0\\ 0 & WTA_{ki}\leq 0 \end{array}$$

For $WTA_{ki}>0$,

(4) $$P(y_{ki}>0)=P(WTA_{ki})=(1/\sigma_{k})\phi [(y_{ki}-\beta_{k}^{T}x_{k})/\sigma_{k}]$$

For $WTA_{ki}\leq 0$, then:

(5) $$P\left(y_{ki}=0\right)=P\left(WTA_{ki}<0\right)=P\left(\varepsilon_{ki}<-\beta_{k}^{T}x_{k}\right)=\Phi \left(-\beta_{k}^{T}x_{k}/\sigma_{k}\right)=1-\Phi \left(\beta_{k}^{T}x_{k}/\sigma_{k}\right)$$

The likelihood function for i is:

(6) $$\begin{array}{ll} L_{ik}(\beta_{k},\sigma_{k})= & 1(y_{ki}=0)\log\left[1-\Phi (\beta_{k}^{T}x_{k}/\sigma_{k})\right]\\ & +1(y_{ki}>0)\log\left\{\left(1/\sigma_{k}\right)\phi \left[\left(y_{ki}-\beta_{k}^{T}x_{k}\right)/\sigma_{k}\right]\right\} \end{array}$$

#### 3.2.2. Variable Selection

Variables were designed based on the above model. In order to analyze the effects of different information on consumer WTA, we denoted AWTA and BWTA, WTA+ and WTA−, and WTA+− and WTA−+, to represent “WTA without information treatment”, “WTA under positive- or negative-information treatment”, and “WTA under both positive- and negative-information treatment”, respectively. Specifically, AWTA, WTA+, WTA+−, are the mean bids of trial 1 to 3, 4 to 6, and 7 to 9 in Group A participants, and BWTA, WTA−, WTA−+, are the mean bids of trial 1 to 3, 4 to 6, and 7 to 9 in Group B participants. Previous studies show that consumers’ knowledge and cognitive capacity are the main factors affecting their perception and WTA [41]. Therefore, we added consumers’ knowledge about additives, consumers’ satisfaction of food safety, and care about food safety as the independent variables. The definition and assignment of variables are shown in Table 3.

## 4. Results

### 4.1. Statistical Description of Participants

#### 4.1.1. Demographics

A total of 310 participants were recruited, 298 samples were obtained with an efficient rate of 96.12 percent. Among the 298 valid samples, there are 150 participants in Group A and 148 participants in Group B. As shown in Table 4, the sample population was predominantly females aged between 25 and 44 years, with a family size of three to five. Moreover, 61.07 percent of participants had children that were younger than the age of 18 in their families, 48.99 percent were graduates of junior college or higher, and 45.64 percent had an average monthly family income of more than 6000 yuan.

#### 4.1.2. Attitude Towards Food Safety and Knowledge of Additives

Table 5 shows that 95.97 percent of participants expressed concern about food safety, but nearly 80 percent were not satisfied with the current food-safety situation. For the information of food additives, 41 percent of the participants knew little about food additives and most of them did not believe the information on food-packaging labels. Nearly 50 percent of participants accessed information on food additives through online media.

#### 4.1.3. Information and Participant Bids

We conducted the auction using multiple trials to allow participants to incorporate food-additive information into their valuations. As shown in Table 6, nine trials were conducted both for Group A and Group B participants.

At the beginning, there were only small differences in the WTA of both groups in the first three trials where no information on additives had been provided. After positive information on additives was provided, the WTA of Group A showed a slight decline in the fourth to sixth trials, with an average decrease of 4.63 percent. In contrast, after negative information was provided, the WTA began to increase rapidly in the seventh trial. The average increase was 76.70 percent, which was significantly greater than the decrease rate in WTA after positive information was provided. For Group B, after negative information was provided, the WTA began to increase rapidly in the fourth trial, with an average increase of 87.30 percent. After positive information was provided, the WTA began to decrease in the seventh trial, with a decrease of 18.08 percent. This was obviously smaller than the increase after negative information was provided. In summary, although the WTA of Group A decreased to some extent after providing positive information, the negative information that was provided later played a dominant role in influencing their WTA. The final average WTA increased from 2.16 yuan to 3.64 yuan, an increase of 68.52 percent. For Group B, the provision of positive information offset some of the impact of the negative information, resulting in a significant decrease in the WTA. However, the WTA still increased by 53.44 percent overall.

Therefore, although the WTA differed with the order in which information was provided, negative information had greater impact on WTA than positive information. Consumers had a significant negativity bias in food-additive risk information. They tended to place more weight on negative product information. Negative information caused consumers to lower their product evaluations more than they did for positive information. Thus, higher compensation was required to compensate for the loss of value. This is consistent with the findings of Siegrist and Cvetkovich [42], Fox, Hayes, and Shogren [26], and Lin and Shih [43]. To identify whether the bids were stable over the nine trials, we also divided the mean bids by the standard deviation in each trial, and the result shows that the bids tend to stabilize in Trials 7 and 8.

### 4.2. Model Results

A Tobit model was established to analyze the main factors influencing consumers’ WTA for orange juice containing additives under different information treatment using STATA 11.0 (StataCorp, College Station, TX, USA). The results are shown in Table 7, Table 8 and Table 9.

According to the parameter estimation results of the no-information treatment (Table 7), age, education, income, satisfaction of food safety, and knowledge of food additives are the main factors influencing consumers’ WTA for orange juice containing additives. Results of Group A show that, when compared with consumers younger than 25 years, the WTA is 0.5188 yuan higher for those aged 60 years and older. Consumers with high school or vocational high school (LEDU), and junior college or undergraduate (MEDU) education have significantly higher WTA than those with education below high school. WTA of consumers with annual family income above 100 thousand yuan (HINCOM) is 0.2772 yuan lower than those with income of less than 60 thousand yuan. Consumers with a medium level of knowledge (MKNOW) about food additives and those with high knowledge (HKNOW) have a higher WTA than those with no knowledge. Results of Group B also show that age is an important factor that affects consumers’ WTA. WTA of consumers who are aged 26 to 45 years (LAGE), 46 to 60 years (MAGE), and over 60 years (HAGE) are significantly higher than those younger than 25 years old. Their WTA are 0.8797, 0.4628, and 1.4421 yuan higher, respectively. Although coefficients of education in Group B do not pass the significance test, the signs are consistent with the results in Group A. As in Group A, consumers with higher income have lower WTA as compared with consumers whose annual family income is less than 60 thousand yuan, WTA is 0.4286 yuan lower for HINCOM consumers. Food-safety satisfaction (SATISF) is also an important factor affecting consumers’ WTA in Group B. Consumers who are satisfied with food safety have a WTA of 0.4070 yuan lower than those who are unsatisfied with food safety.

Under positive information treatment, age, income, and knowledge of food additives are still the main factors influencing consumers’ WTA in Group A (Table 8). Consumers aged above 60 years old (HAGE) still have a higher WTA than those that are younger than 25 years old. After providing positive information, HINCOM consumers need less compensation. Besides, under positive information treatment, WTA of SATISF consumers is also significantly reduced. MKNOW and HKNOW still have a higher WTA than those with no knowledge, but education is no longer the most significant factor that affects consumers WTA.

Under negative information treatment, factors that influenced consumers’ WTA in Group B change greatly (Table 8). Firstly, after receiving negative information, WTA of females increases significantly; females’ WTA is 0.6289 yuan more than that of males. Secondly, WTA of those less-educated consumers increases significantly under negative information treatment. Compared with consumers without high-school education, WTA of LEDU and MEDU consumers increase by 0.7974 and 0.4510 yuan, respectively. Thirdly, affected by negative information, WTA of SATISF consumers is no longer significantly reduced. Finally, family income is not a significant factor affecting consumers WTA anymore. However, age is still a main factor affecting consumers’ WTA. When compared with consumers aged less than 25 years, WTA was 2.0974, 1.3716, and 2.5587 yuan higher for LAGE, MAGE, and HAGE consumers, respectively, which is much higher than those without information intervention.

Under both positive and negative information treatment for Group A consumers (Table 9), results show that knowledge of food additives is still a significant factor affecting consumers WTA. HAGE still have a higher WTA than those younger than 25 years old. In addition, MAGE consumers significantly increase their WTA. However, influenced by negative information, the annual income of households is no more a main factor affecting consumers’ WTA, and WTA of consumers who are satisfied with food safety is no longer significantly lower than those who are not satisfied with food safety.

Under both negative and positive information treatment, female and age are still the main factors affecting consumers’ WTA in Group B (Table 9). WTA of females is 0.3953 yuan higher than males, which lower than the WTA only affected by negative information. Affected by positive information, LAGE, MAGE, and HAGE consumers slightly reduced their compensation. Their WTA are only 1.7048, 1.3095, and 1.2522 yuan, respectively, higher than that of consumers younger than 25 years old. However, education is no more a factor affects consumers’ WTA.

## 5. Discussion

The results show that variables of age (HAGE), education (MEDU, LEDU), income (HINCOM), satisfaction of food safety (SATISF), and knowledge of food additives (LKNOW, HKNOW) are the main factors that affect consumers’ WTA when there is no information treatment. However, when consumers are provided with different types of additives information, the main factors that affect consumers’ WTA change.

First, females are more sensitive to information on additives than males, and they are more susceptible to negative information. Lin and Chen [44] found that females are more likely than males to focus on comprehensive information and to carefully process information that suits their own interests. Sun [45] stated that the level of information processing determines the level of risk perception, and thus results in gender differences in risk perception.

Second, consumers’ WTA increased with age, despite the negative or positive information provided. The possible reason for this is that consumers are more concerned about their own health as they get older, so they need a higher WTA. This finding is similar to the conclusions of Mou and Lin [46]. Although the higher WTA does not change as information provided, information interventions change the increase rate of WTA: negative information aggravates consumers’ risk perception, thus leading to a higher increase rate of WTA. Positive information partially offsets the risk of food additives, thus reducing the increase rate of WTA.

Third, the influences of education on consumers’ WTA are complicated: HEDU do not affect consumers’ WTA whether there is information treatment or not. Consumers with postgraduate education and above are more rational about the risk perception of food additives, and they are not easily affected by information treatment. However, consumers with medium education, on the one hand, have higher acceptance of external information than those without education. On the other hand, they are vulnerable to the uncertainty of information than those with postgraduate education and above; therefore, their WTA change greatly when provided with positive or negative information.

Fourth, MKNOW and HKNOW consumers have a higher WTA. This finding is similar to the conclusions of Liu et al. [47]. There are three possible explanations for this: (a) Knowledge of consumers who said they knew about food additives is subjective, which is different from objective knowledge [20]. Subjective knowledge might not be comprehensive or accurate. Therefore, influenced by their subjective knowledge, consumers’ WTA might be higher than those without knowledge of food additives. (b) Positive or negative information received in the experiment might conflict with consumers’ previous understanding, which increased the uncertainty of the information, as well as their risk perceptions, thus resulting in a higher WTA. (c) The complexity of food additives could lead to poor dissemination or even to intentional distortion of relevant information, thus triggering consumers’ risk perception of food additives. As a result, their WTA increases after receiving positive or negative information. This study supports the findings of Slovic and Miles et al. [48,49] that uncertain information and unknown knowledge aggravate public risk perception and that uncertain information is more likely to aggravate risk perception than unknown knowledge.

Fifth, food-safety satisfaction is an important factor affecting consumers’ WTA. Consumers who are satisfied with food safety have significantly lower WTA than those who are not satisfied with food safety. This finding is supported by previous studies like Worsfold [50]. The reason for this is that consumers who are satisfied with food safety might have confidence incurrent food safety situation, and therefore, they need less compensation to accept food with additives. However, negative information will reduce consumers’ satisfaction, and this negative perception cannot be offset by providing positive information.

## 6. Conclusions

Consumers in China alter their purchase behaviors based on their concerns about food safety [51]. In this study, Chinese consumer willingness to accept compensation to exchange freshly squeezed orange juice without additives for orange juice containing additives was examined via a random *n*th-price auction. On this basis, the respective effects of positive and negative information regarding orange-juice additives on consumers’ risk perception were investigated while using the Tobit model. We find a negativity bias in consumers, with negative information on orange-juice additives being given more weight than positive information. A higher WTA is reported by consumers with additive knowledge compared with those without. WTA of consumers with medium education changes greatly under information intervention. We also find a gender difference in risk perception. Consumers’ WTA increases with age and decreases with levels of satisfaction with food safety.

How to solve widespread consumer worries about food safety, and guide consumers to correctly understand food additives, rationally respond to food-safety incidents, and rebuild confidence in the Chinese food industry are urgent problems that need to be solved by the Chinese government. Food-safety risk communication is an effective way to eliminate food-safety scares. The government should pay more attention to the monitoring of food production when there is a high degree of risk and strong social concerns. By doing so, the likelihood of food-safety incidents occurring could be reduced to a minimal level, and the spread of related social-media rumors could be eliminated at the source. Food-safety rumors and false information have a great effect on consumers’ risk perception; the government should prevent the media, especially online public opinion, from misleading consumers at the source through legal means. Uncertain food-safety information aggravates consumers’ risk perception, and consumers with some knowledge about additives have a higher WTA than those without knowledge about additives. Therefore, the Chinese government must first establish an open and transparent information-dissemination mechanism to ensure timely, comprehensive, and accurate disclosure of food-safety information; avoid contradiction between information disclosed by different departments; and, in particular, avoid the traditional policies of obscurantism or ignoring public questioning.

This study had several limitations. First, subjects (participants) are merely from Suzhou, China. Future studies could conduct similar study in a larger range, in terms of both subject group characteristics and the geographic region included. Second, further studies can make in-depth investigations on the distinction between objective versus subjective knowledge of consumers and its influences on food safety risk perception and their willingness to accept. Third, it is essential for future studies to verify how respondents conceptualize food safety and food additives in detail, which has just been done limitedly here, in order to provide more meaningful results. Indeed, this issue could be an independent study to focus and improve.

## Figures and Tables

**Table 1:** The recruitment plans and information release order.

Group	The Rrder of Information	Number of Participants	Recruitment Place
A1	No information, positive information, negative information	38	Auchan Jinji Lake
B1	No information, negative information, positive information	41	Auchan Jinji Lake
A2	No information, positive information, negative information	35	WalMart Nanmen
B2	No information, negative information, positive information	43	WalMart Nanmen
A3	No information, positive information, negative information	32	RtMart Heshan Road
B3	No information, negative information, positive information	47	RtMart Heshan Road
A4	No information, positive information, negative information	36	Carrefour Wanda plaza
B4	No information, negative information, positive information	38	Carrefour Wanda plaza

Note: Number oftotal recruited participants, *n* = 310.

**Table 2:** Different types of information provided in experimental auctions.

Positive Information	Negative Information
Strictly follow the standard, and use food additives approved by authorities that have no safety risks.	As food additives are not natural ingredients, their long-term intake, even in small amounts, may cause harm to the human body.
Food additives, such as sweeteners and tartrazine, can help improve the color, smell, and taste of orange juice by enhancing its flavor and brightening its color.	A long-term rat-feeding trial conducted in 1969 showed that a high concentration of sodium cyclamate mixed with saccharin could lead to bladder cancer in rats.
Preservatives added to orange juice can help extend its shelf life, and keep it fresh and antiseptic.	When the experimental rats were fed with 8 percent feed containing benzoic acid, a type of preservative, their livers and kidneys generally showed pathological changes after 90 days, and more than half of the rats soon died.
Preservatives commonly used in orange juice, such as sodium benzoate and potassium sorbate, can not only keep orange juice from deteriorating, but can also kill pathogens and other microbes during juice processing, and thus enhance the quality of the juice.	Colorants, such as tartrazine and sunset yellow commonly found in orange juice, are synthetic pigments. Once they enter the human body, a large number of the body’s detoxification substances are used, which not only interferes with the body’s normal functions, but might also lead to hepatitis, calculus, diarrhea, and dyspepsia.

**Table 3:** The variable index determination for the Tobit model.

Variable	Classification Index	Mean	Standard Deviation
Willingness to accept (WTA) without information treatment	Mean bids of trial 1 to 3 in Group A (AWTA).	2.16	1.68
	Mean bids of trial 1 to 3 in Group B (BWTA).	1.89	1.36
WTA under positive information	Mean bids of trial 4 to 6 in Group A (WTA+).	2.06	1.67
WTA under negative information	Mean bids of trial 4 to 6 in Group B (WTA−).	3.54	2.32
WTA under positive and negative information	Mean bids of trial 7 to 9 in Group A (WTA+−).	3.64	2.67
WTA under negative and positive information	Mean bids of trial 7 to 9 in Group B (WTA−+).	2.57	1.60
Female	No = 0, Yes = 1 (FEMALE)	0.52	0.50
Age 26 to 45	No = 0, Yes = 1 (LAGE)	0.55	0.50
Age 46 to 60	No = 0, Yes = 1 (MAGE)	0.12	0.33
Age above 60	No = 0, Yes = 1 (HAGE)	0.07	0.25
High school or vocational high school	No = 0, Yes = 1 (LEDU)	0.34	0.48
Junior college or above	No = 0, Yes = 1 (MEDU)	0.40	0.49
Master or above	No = 0, Yes = 1 (HEDU)	0.06	0.24
Yearly family income ¥60–100 thousand	No = 0, Yes = 1 (MINCOM)	0.13	0.34
Yearly family income above ¥100 thousand	No = 0, Yes = 1 (HINCOM)	0.35	0.47
Inclusion of dependent children in the family	No = 0, Yes = 1 (KID)	0.62	0.53
Medium knowledge about additives	No = 0, Yes = 1 (MKNOW)	0.42	0.49
High knowledge about additives	No = 0, Yes = 1 (HKNOW)	0.55	0.50
Satisfaction of food safety	No = 0, Yes = 1 (SATISF)	0.23	0.42
Care about food safety	No = 0, Yes = 1 (CARE)	0.96	0.20

**Table 4:** The demographic characteristic of participants.

Variables	Categories	Sample Size	Percentage (%)
Gender	Male	144	48.32
	Female	154	51.68
Age	Under 18	2	0.67
	18–25	76	25.50
	26–45	164	55.03
	45–60	36	12.08
	Above 60	20	6.72
Education	Elementary school	10	3.36
	Middle school	38	12.75
	High school	104	34.90
	Junior collage	82	27.52
	University	56	18.79
	Postgraduate	8	2.68
Family size	1	2	0.67
	2	24	8.05
	3	118	39.60
	4	36	12.08
	More than 5	118	39.60
Yearly family income	Under ¥30 thousand ^1^	26	8.73
	¥30–60 thousand	40	13.42
	¥60–100 thousand	102	34.23
	¥100–150 thousand	74	24.83
	Above ¥150 thousand	56	18.79

^1^ ¥ is CNY, 1 CNY is equal to 0.145 USD.

**Table 5:** Food safety and information about additives.

Variables	Categories	Sample Size	Percentage (%)
Food safety concerns	No	12	4.03
	Yes	286	95.97
The satisfaction level of food safety	Unsatisfied	230	77.18
	Somewhat satisfied	60	20.13
	Very satisfied	8	2.69
The level of trust in food labels.	Low trust level	160	53.69
	Neutral	78	26.17
	High trust level	60	20.14
Knowledge about food additives.	None	124	41.61
	A little	164	55.03
	Sufficient	10	3.36
Source of information about food additives.	Paper or magazine	34	11.41
	TV or broadcast	86	28.86
	Web media	142	47.65
	Authorities or official information	10	3.36
	Scholars or specialists	12	4.03
	Families or friends	14	4.69

**Table 6:** Mean bids by trial for Group A and Group B.

**Group A**	**No Information**				**Positive Information**				**Negative Information**			
	**Bids in Trial 1**	**Bids in Trial 2**	**Bids in Trial 3**	**Mean**	**Bids in Trial 4**	**Bids in Trial 5**	**Bids in Trial 6**	**Mean**	**Bids in Trial 7**	**Bids in Trial 8**	**Bids in Trial 9**	**Mean**
Mean	2.16	2.12	2.21	2.16	2.13	2.05	2.01	2.06	3.53	3.63	3.77	3.64
Median	1.50	1.80	1.80	–	2.00	2.00	2.00	–	2.80	2.90	3.00	–
S.D.	1.84	1.63	1.58	–	1.74	1.65	1.62	–	2.61	2.66	2.73	–
Mean/S.D.	1.17	1.30	1.40	–	1.22	1.24	1.24	–	1.35	1.36	1.38	–
**Group B**	**No Information**				**Negative Information**				**Positive Information**			
	**Bids in Trial 1**	**Bids in Trial 2**	**Bids in Trial 3**	**Mean**	**Bids in Trial 4**	**Bids in Trial 5**	**Bids in Trial 6**	**Mean**	**Bids in Trial 7**	**Bids in Trial 8**	**Bids in Trial 9**	**Mean**
Mean	1.98	1.76	1.92	1.89	3.44	3.56	3.62	3.54	2.67	2.54	2.50	2.57
Median	1.80	1.50	1.8	–	3.20	3.00	3.50	–	2.00	2.00	2.00	–
S.D.	1.42	1.20	1.56	–	2.19	2.35	2.36	–	1.67	1.60	1.54	–
Mean/S.D.	1.39	1.46	1.23	–	1.57	1.51	1.53	–	1.60	1.59	1.62	–

**Table 7:** Parameter estimation results of no-information treatment.

Variable	Bids of Trial 1 to 3 in Group A (AWTA)			Bids of Trial 1 to 3 in Group B (BWTA)		
	Coefficient	Std. Err	*P* > |*t*|	Coefficient	Std. Err	*P* > |*t*|
FEMALE	0.0095	0.1272	0.9400	0.1274	0.1239	0.3060
LAGE	0.1736	0.2342	0.4600	0.8797 ***	0.2211	0.0000
MAGE	0.1944	0.3383	0.5667	0.4628 *	0.2761	0.0959
HAGE	0.5188 **	0.2603	0.0481	1.4421 ***	0.3116	0.0000
LEDU	0.5456 ***	0.1961	0.0060	0.0910	0.1970	0.6448
MEDU	0.6035 ***	0.1631	0.0000	0.2033	0.2196	0.3562
HEDU	−0.0622	0.1753	0.7234	−0.3669	0.2938	0.2133
MINCOM	−0.0276	0.1883	0.8835	−0.1279	0.1651	0.4400
HINCOM	−0.2772 **	0.1387	0.0480	−0.4286 ***	0.1421	0.0030
KID	0.1324	0.1590	0.4069	0.1985	0.1970	0.3145
CARE	0.1908	0.2885	0.5100	0.6325	0.4539	0.1664
SATISF	−0.0552	0.1659	0.7400	−0.4070 ***	0.1490	0.0070
LKNOW	1.0407 ***	0.2529	0.0000	−0.1371	0.3113	0.6600
HKNOW	1.6495 ***	0.3023	0.0000	−0.2872	0.3265	0.3809
CONSTANT	0.0261	0.4379	0.9348	2.4098	0.6157	0.0000
*σ*	0.3604	0.0526	—	0.6249	0.0491	—
*N*	150			148		

*, ** and *** denote significance at 10%, 5%, and 1% levels, respectively.

**Table 8:** Parameter estimation results under single information treatment.

Variable	Bids of Trial 4 to 6 in Group A (WTA+)			Bids of Trial 4 to 6 in Group B (WTA−)		
	Coefficient	Std. Err	*P* > |*t*|	Coefficient	Std. Err	*P* > |*t*|
FEMALE	−0.3423	0.2677	0.2031	0.6289 **	0.2579	0.0160
LAGE	0.3927	0.4563	0.6349	2.0974 ***	0.5586	0.0000
MAGE	0.3714	0.5925	0.5322	1.3716 **	0.5686	0.0170
HAGE	0.8613 *	0.8260	0.0608	2.5587 ***	0.4518	0.0000
LEDU	−0.0280	0.5224	0.9671	0.7974 **	0.5250	0.0130
MEDU	−0.0101	0.4076	0.9800	0.4510 **	0.4526	0.0321
HEDU	−0.0081	0.4129	0.9843	0.1459	0.4045	0.7189
MINCOM	−0.2718	0.2852	0.3417	0.1213	0.2975	0.6843
HINCOM	−0.8343 *	0.4724	0.0800	−0.2281	0.3471	0.5124
KID	0.5219	0.3597	0.1492	0.4949	0.4026	0.2207
CARE	0.2619	0.5901	0.6579	1.3097	0.9628	0.1764
SATISF	−0.7211 *	0.4200	0.0880	−0.4307	0.3079	0.1643
LKNOW	2.3243 ***	0.8748	0.0090	0.4658	0.6531	0.4772
HKNOW	3.1463 ***	0.8691	0.0000	−0.2884	0.6846	0.6738
CONSTANT	−0.0997	1.2114	0.9347	3.5770 ***	1.3053	0.0070
*σ*	1.4023	0.0871	—	1.3634	0.0948	—
*N*	150			148		

*, ** and *** denote significance at 10%, 5%, and 1% levels, respectively.

**Table 9:** Parameter estimation results for two types of information treatment.

Variable	Bids of Trial 7 to 9 in Group A (WTA+−)			Bids of Trial 7 to 9 in Group B (WTA−+)		
	Coefficient	Std. Err	*P* > |*t*|	Coefficient	Std. Err	*P* > |*t*|
FEMALE	−0.2267	0.3129	0.4700	0.3953 **	0.1994	0.0488
LAGE	0.3544	0.5300	0.5049	1.7048 ***	0.3471	0.0000
MAGE	0.8519 **	0.3366	0.0130	1.3095 ***	0.4397	0.0030
HAGE	0.8885 **	0.5323	0.0970	1.2522 ***	0.4371	0.0050
LEDU	0.3853	0.4797	0.4232	0.4213	0.3118	0.1793
MEDU	0.2030	0.4723	0.6681	0.4525	0.3466	0.1324
HEDU	0.7920	0.6125	0.1983	0.5046	0.4687	0.2825
MINCOM	0.2377	0.6930	0.7321	0.0028	0.2734	0.9923
HINCOM	−0.4824	0.9544	0.6139	0.6512	0.2301	0.1050
KID	0.0470	0.4186	0.9107	0.0726	0.3065	0.8134
CARE	0.2812	0.6733	0.6773	0.7705	0.6992	0.2719
SATISF	−0.1353	0.1297	0.2981	−0.0035	0.2422	0.9983
LKNOW	2.5049 ***	0.9179	0.0070	0.4893	0.4860	0.3162
HKNOW	3.5414 ***	0.9123	0.0000	0.6500	0.5059	0.2010
CONSTANT	0.7738	1.3129	0.5570	1.7429 **	0.9704	0.0747
*σ*	1.5921	0.1137	—	1.0863	0.0675	—
*N*	150			148		

*, ** and *** denote significance at 10%, 5%, and 1% levels, respectively.
